# The Effect of Bad News and CEO Apology of Corporate on User Responses in Social Media

**DOI:** 10.1371/journal.pone.0126358

**Published:** 2015-05-07

**Authors:** Hoh Kim, Jaram Park, Meeyoung Cha, Jaeseung Jeong

**Affiliations:** 1 Graduate School of Culture Technology, Korea Advanced Institute of Science and Technology (KAIST), 305–701, Daejeon, Republic of Korea; 2 Department of Bio and Brain Engineering, Korea Advanced Institute of Science and Technology (KAIST), 305–701, Daejeon, Republic of Korea; Xiamen University, CHINA

## Abstract

While social media has become an important platform for social reputation, the emotional responses of users toward bad news have not been investigated thoroughly. We analyzed a total of 20,773 Twitter messages by 15,513 users to assess the influence of bad news and public apology in social media. Based on both computerized, quantitative sentiment analysis and in-depth qualitative analysis, we found that rapid public apology effectively and immediately reduced the level of negative sentiment, where the degree of change in sentiments differed by the type of interactions users engaged in. The majority of users who directly conversed with corporate representatives on the new media were not typical consumers, but experts and practitioners. We extend the existing cognitive model and suggest the audiences’ psychological reaction model to describe the information processing process during and after an organizational crisis and response. We also discuss various measures through which companies can respond to a crisis properly in social media in a fashion that is different from conventional mass media.

## Introduction

Social media has recently become a game changer in how modern businesses operate [1]. Social media or social network sites are Web-based services, where users can publicly or privately share information and opinions with one another. Social media users build relationship as they share common interests or views on certain issues, from movies and books to politics and to celebrities [2,3]. Customers have ever more power to express their voice, while corporate reputations can be easily at stake by bad news and rumors that spread rapidly in social media. The unpredictable, cascading feature of corporate crisis in social media is at its butterfly effect: It likely starts with a single message in a network but spread to global-scale eventually leading to a devastating consequence for the business at risk. One example is the Papa John’s Pizza Crisis in 2012 that started with a single message on Twitter from a Korean-American customer in New York, who was furious to find a note “lady chinky eyes” written on her receipt by a cashier. Through her negative recounting of the event on Twitter, a local newspaper picked up the story and the news went to national newspapers as well as international news media like CNN within a few days. Papa John’s fired the cashier, sent out a public apology, and even the Papa John’s branch in Korea apologized to Korean customers [4].

For individuals and companies that care about their reputations, it is critical to know how to respond and cope with such crisis in social media. Many researchers on public relations have hence studied crisis communication through new media [4–7], yet several main challenges still remain in the field of crisis communication. One such challenge is at the lack of solid data-driven research methodology. A systematic review of crisis communication research literature finds that only 13% of the research articles reviewed proposed research questions or theory-based hypotheses and only about 26% had properly reported their research methods [8,9].

Another challenge is that the audience and their emotions have been neglected as many studies have commonly identified [10,11]. Most prior studies have focused on analyzing the content of messages rather than emotions [11] and have provided the views of corporations rather than the audience [12]. Consequently, the key question of how consumers’ emotions are influenced by corporate crisis response strategies remains unanswered [13], and a deeper understanding of the audience behavior and emotion is critically required. Therefore, the current study was designed to investigate the real-time interaction behaviors of new media users on a popular real crisis. We chose a major micro-blogging service, Twitter, for this study, because the service is known for its ability to spread viral news rapidly and to influence the audience with the stories. The influence relationship in Twitter is explicitly determined by the follow links, which indicates who is listening to whom. In Twitter, both opinion leaders and traditional media like newspapers and magazines play an important role as an influencer [14]. Recently, Twitter has drawn much attention as a media of political movement in Iran and Egypt [15] and a socio-economic indicator of election results [16], stock prices [17], and natural disasters [18]. For research data acquisition, Twitter provides Application Programming Interface (API) that allows large-scale data gathering. Considering its active nature of interaction by users and data accessibility, Twitter is one of the most appropriate channels to investigate the behavior of new media users on notable social events.

In the current study, we attempted to address the following key questions: First, how do Twitter users respond to negative news on corporate crisis and public apologies in social media? Second, do the users’ sentiments toward a crisis event differ depending on the types of interaction they engage in social media? Third, what are the types of social media response upon a corporate crisis and how do they spread depending on the type? Fourth, how do the tweets of different types (i.e. bad news, apology, commentary) diffuse in Twitter? Fifth, upon a corporate crisis, to what extent do customers in social media show negative responses that could impact product sales in future? Lastly, what is the role of a corporate representative account in social media upon a crisis and who communicate with the corporate account?

To answer these questions, we conducted an in-depth and systematic data analysis on a real event that spawns heavy reactions in Twitter, the Domino’s Pizza Crisis in 2009. We chose this event in this study for a few of reasons: First, this case received great attention in both traditional media and social media, and produced a large amount of conversations in social media. In fact, this crisis event initially spread out in Twitter and hence produced sufficient amount of Twitter users’ response data. Furthermore, this case included a proper public apology from the company involved in this crisis event, which was also transmitted and propagated within Twitter. Finally, the company of crisis set up a corporate account in Twitter to converse with potential customers and manage the aftermath of crisis directly, which allows us to investigate the effect of direct and indirect interactions with potential customers on group sentiment in Twitter.

The crisis started on 13 April 2009 at the Domino’s Pizza branch in Conover, North Carolina, with a crude video showing two employees’ vulgar acts while making sandwiches in the restaurant’s kitchen, which they later posted on YouTube. One employee was seen inserting cheese into his nose, spreading nasal mucus on the sandwiches violating various health-code standards; the other employee provided commentary. Within two days, the video received more than 500,000 views and sparked shocked and angry comments throughout social media. At the same time, Mr. Patrick Doyle, the president of Domino’s Pizza, responded with a YouTube video directly expressing his deep regret about the incident within two days. In addition, Domino’s responded to the public and furthered shared the apology video by opening up a new Twitter account, named @dpzinfo. The crisis was finally resolved as the company prepared a civil lawsuit and the two employees faced felony charges for attempting to deliver prohibited food to customers.

The Domino’s Pizza incident is one of the first events that epitomizes large-scale social media crisis. The crisis event not only went viral among social media users, but also led to negative consequences in terms of reputation damage to the company. According to the BrandIndex measurement scores by the research firm YouGov, Domino’s score significantly declined from 20 to -1 within three days from the crisis event [19]. Furthermore, the stock price of Domino’s Pizza dropped 10% in the first week of the crisis [20]. To deeper understand the reaction of social media users toward a popular crisis, we gathered to analyze a total of 20,773 tweets by 15,513 users that were relevant to the Domino’s Pizza incident, drawn from the complete pool of 1.7 billion tweets and 54 million users of Twitter at the time of summer 2009.

The current study potentially makes the following contributions. First, to the best of our knowledge, we analyzed the largest amount of data in social media to assess the real-time reactions and behaviors of users upon a corporate crisis. Until now, crisis communication studies could only analyze the post-crisis opinions through surveys taken over a small sample due to the difficulty in gathering data. Second, the current study attempted to provide a unique view on the effect of corporate apology during a crisis event. In the past, many companies covered up their wrongdoings rather than acknowledged them, and even if they did so and apologized publicly, it was only after a long time. However, as social media contribute to diffusing bad news rapidly and widely, companies appeared to respond to corporate crises more quickly and to apologize publicly through social media [21]. In this respect, this study addressed a significant question of how a rapid public apology took place in social media and of what was its effect in changing the sentiments of users.

Third, computational social science research utilizes various big data approaches to better understand social phenomena [3]. Since often the age, gender, and ethnicity information about users is not available in social media, researchers have relied on geo-location information written on profiles or facial markers embedded in photos of users to infer demographics [22–24]. The vast majority of social network research has treated information propagation "content agnostic". However, information in reality may diffuse with different patterns according to their types and context. In this respect, the novelty of this paper lies at treating bad news, facts, and apology separately. We assume these types of information propagate under different mechanisms and utilize a large amount of social media data to prove this hypothesis. Finally, the current study analyzed the specific group of Twitter users who directly communicate with the corporate representatives during a crisis. Before the social media era, companies announced their position statement in crisis and journalists reported crisis events, which were both one-way communications. Nowadays, as potential consumers produce content real-time and engage in two-way communications in social media, there is growing importance of two-way conversations. In particular, companies can directly communicate with potential customers even under crisis situations. However, it has been unclear who directly talks to responsible companies in crisis and what their voices are. The current study attempted to investigate who they are in social media.

## Data and Methods

### Twitter Data

To examine the spreading pattern of any piece of information in the global network, simply analyzing a sample of tweets would create biases in the measured sentiments and gaps in the information propagation patterns (e.g., the spreading pattern will be segmented). Therefore, access to the entire tweet posts and the complete social network topology is essential. In the current study, we had access to a near-complete Twitter dataset from a previous work [25]. The data consisted of information of 54 million users, 1.9 billion social links, and 1.7 billion tweets. The follow links were based on a topology snapshot taken in the summer of 2009, a few months after the Domino’s incident. The 1.7 billion tweets included all public tweets that were ever posted by the 54 million users since the start of Twitter. Each tweet entry contained the tweet content, the corresponding time stamp, and the user’s screen name.

All tweets that contained the word “domino” over an eight-day period from 13–20 April 2009 were selected for analysis. A total of 19,328 tweets were found in this manner from a corpus of complete Twitter dataset. This selection method incurred both false positives (i.e., including tweets irrelevant to the event although containing the keyword) and true negatives (i.e., missing tweets relevant to the event that do not contain the keyword). To minimize this error, we relied on the fact that more than half (60%) of tweets on public events included a URL or a web link. We focused on URLs mentioned more than ten times, for whom we manually verified their relevance to the Domino’s crisis, and added those tweets containing the corresponding URLs (but not necessarily containing the keyword) as valid data to be analyzed. As a result, 1,445 true negative tweets were found from the complete Twitter corpus, entailing a final set of 20,773 tweets to be analyzed. A complete dataset can be found in S1 Dataset.

### Quantitative sentiment analysis—Linguistic Inquiry and Word Count (LIWC)

To determine whether each tweet of attitudes format was positive or negative, we used Linguistic Inquiry and Word Count (LIWC), which is a transparent text analysis program that counts words in psychologically meaningful categories. LIWC is a widespread program that social media researchers utilize for sentiment analysis [16,26,27]. Empirical studies have demonstrated that LIWC is useful in various experimental settings for detecting the meaning of words including differences in attention, emotion, sociality, and logic [28]. In this study, sentiments and attitudes were used interchangeably.

In this study, we limited our focus to the affective psychological process among the categories that LIWC supports. In particular, we analyzed the frequency of tweets corresponding to the Positive Affect (PA) and the Negative Affect (NA) within the affective psychological process. Note that these two affects, PA and NA, are not always opposite to each other, but rather the levels of positive affect and negative affect may be independent [27]. Utilizing tweets for sentiment analysis has certain limitations; first because of the unstructured fashion the text is written and second because of the short text length. According to the providers of the LIWC, more than 50 words are required for accurate analysis, which indicates that tweets with fewer than 50 words could show abnormal sentiment scores. Because of these limitations, we could not conduct a fine-grained analysis to infer sentiment of a single tweet or that of an individual. Furthermore, given that LIWC analysis simply calculates sentiment scores based on word frequency, we could not detect subtle mood changes or differences, for instance, the tone of voice. In order to address limitations of such quantitative approach, we adopted an alternative method to investigate the sentiment propagation [27].

### Qualitative analysis—Open coding

Qualitative analysis was conducted to gain contextual understanding of the tweet content, in addition to the insights gained from quantitative analysis of data [29]. In qualitative analysis, the coding scheme is developed for a limited size of data utilizing various methods such as the grounded theory [30]. In this work, the open coding approach, an analysis method that specifically addresses the naming and categorizing phenomena through thorough data analysis, was used so that relevant categories could emerge. [31]. Once the open coding scheme was developed, the first author who had trained in qualitative analysis counted the frequency of each code in data.

A total of 860 Twitter conversations from two peak times were sampled for analysis: (1) 395 tweets from 3 to 4 A.M. (based on the Eastern time zone or EST) on April 15^th^ during the peak dissemination time of the prank video and (2) 465 tweets from 8 to 9 P.M. on April 16^th^ during the peak dissemination time of the apology video. We eliminated tweets that seemed irrelevant to the 2009 Domino’s crisis after extensive data review. For example, one tweet said “Picking up Dominos for the fam. Wife called it in. Pepperoni for the kids…”; this tweet was about Domino’s Pizza but was irrelevant to the crisis. Tweets written in language other than English were also eliminated. Furthermore, ambiguous tweets like “Domino Pizza Time” were not included. Content that we considered irrelevant and thus excluded from qualitative analysis were in aggregate 130 tweets (61 from the first peak and 69 from the second peak) out of 860 tweets.

For the analysis of conversation between Twitter users and Domino’s official Twitter account (@dpzinfo), we collected a total of 456 tweets that mention @dpzinfo, and 1159 tweets relevant to the Domino’s Pizza crisis that have been uploaded by 378 Twitter users who mentioned @dpzinfo at least once. For the qualitative analysis, the unit of analysis was each tweet content.

## Results

Table 1 shows the number of Twitter users, tweets, mentions, retweets (RTs), and tweets on the Domino’s case. A distinctive set of 15,513 users posted at least one tweet, which indicates that most users posted only one tweet about the Domino’s incident. Out of all relevant tweets, 4,990 or 24% of the tweets were mentions or replies to other tweets, which were identified by the @username marker in the tweet. Among tweets, 2,673 or 13% were retweets, where the most popular tweet was retweeted more than 500 times. This trend indicates that Twitter users actively conversed about the Domino’s Pizza incident.

**Table 1:** Summary of the Twitter dataset used in this study.

#of Users	# of Tweets	# of Mentions	# of RTs	# of URL(%)
15,513	20,773	4,990	2,673	13,132 (63%)

### (Research Question 1) How do users respond to negative news on corporate crisis and public apologies in social media?

**Fig 1:**
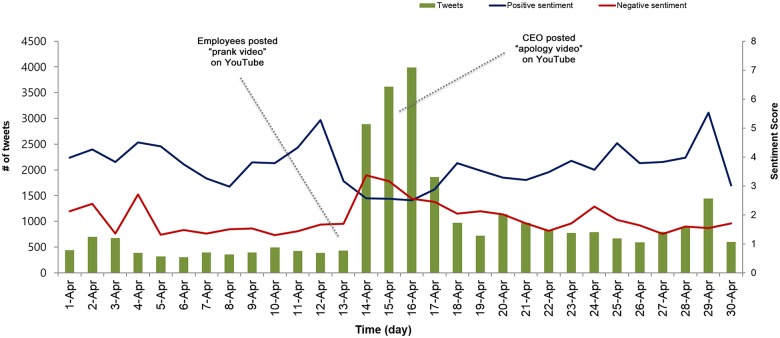
Temporal evolution of the number of tweets per day (green) during April 2009, and the positive (blue) and negative (red) sentiment scores measured by Linguistic Inquiry and Word Count. The Domino's Pizza crisis started on 13th and the public apology by the Domino Pizza CEO was delivered on 15th.

To answer this question, we conducted quantitative and qualitative analyses of tweets on Domino’s Pizza incident. Fig 1 shows the results of the quantitative analysis, where the bar graph indicates the daily number of tweets with the word “domino” during the month of April, 2009. Within a day since the upload of prank video, the number of tweets on Domino’s Pizza rose to over 2500 tweets per day, which is five times larger than in the previous week. Within two days (14 and 15 April), there were already 7,000 tweets on the incident. Over the course of eight days from 13^th^ to 20^th^ April of 2009, an estimated 16,553,169 distinct users (or 30% of all Twitter users) had been potentially exposed to the news, based on the follow link relationship.

In Fig 1, the line graphs overlaid on top of the bar graph show the degree of positive affect (blue line) and negative affect (red line) of the tweets, which was coded automatically from the LIWC analysis. Three days between 14th and 16th were peak days in terms of the number of tweets and were the lowest among days in April in terms of positive sentiments. The negative sentiment was the highest on April 14^th^. Prior to the crisis, Twitter users demonstrated an overall positive affect towards Domino’s Pizza. However, the trend becomes reversed during the three peak days. A randomly chosen set of 10,000 tweets that does not contain the word “Domino” within April 13–20 shows that the levels of positive affect and negative affect were 4.24 and 1.65, respectively compared to tweets containing the word “Domino”.

In addition, Fig 1 depicts the exact times when the amount of conversations and the level of negative sentiment changed; they surged immediately after the dissemination of the prank video on social media and dropped quickly after the dissemination of the public apology. This flux in trend can imply that the apology video was an appropriate and effective response (and a rather fast one—within 48 hours) from the perspective of crisis management.

In qualitative analysis, we attempted to detect major issues that arose in the tweets and found two formats of tweet content: One was without attitudes and the other was with attitudes towards the event. The former indicates having neutral emotion toward the event and the latter indicates either positive or negative evaluation towards the event [32].

Neutral tweets included mere web links without any text or web links with a headline of an external webpage, for instance: *‘Searched Twitter for dominos*: *http*:*//tinyurl*.*com/c4s4lx* *’; ‘See the video*: *http*:*//ping*.*fm/9Eybi* *’*

Also, simple statements or questions and suggestions were also categorized as neutral: ‘*@tabitharay Where was that Dominos located*?*’* It should be noted that as our focus of analysis was Domino’s Pizza, it was considered as attitudes format only when it was about Domino’s Pizza. For example, the following tweets showed positive attitudes but it was not towards Domino’s Pizza, thus should be categorized as neutral: *‘@chrisamichaels*: *@FusionPR*: *Great post by @stevebandrews about Domino's FAIL at crisis communications* *http*:*//is*.*gd/srkf*.*’* During the first peak that appeared immediately following the launch of the prank video, 67 out of the 334 relevant tweets (20.1%) were categorized as neutral, whereas in the second peak (right after the launch of the apology video), 165 out of the 396 relevant tweets (41.7%) were neutral (Table 2).

**Table 2:** Qualitative analysis results of tweets.

	The 1^st^ peak	The 2^nd^ peak
Neutral	67 (20.1%)	165 (41.7%)
Positive opinions	1 (0.3%)	25 (6.3%)
Negative opinions	266 (79.6%)	206 (52%)
Total	334 (100%)	396 (100%)

On the other hand, the attitudes format contained the tweets that had either positive or negative emotions or evaluations towards Domino’s Pizza, where most opinions were negative due to the nature of the event. However, a few Twitter users had positive sentiments towards the crisis; for instance, one said, *“Yes*, *I read the stories about Dominoes on Consumerist today*. *No*, *that didn’t stop me from just ordering a philly cheesesteak pizza*.*”* Furthermore, certain tweets contained both positive and negative sentiments based on automatic tagging, and hence had to be analyzed manually to judge the major sentiment. In the following example, the tweet contains positive sentiment at the beginning but ends with regret: “*liking the response by dominos…wish he would have looked at the camera tho* *http*:*//tinyurl*.*com/c8dju3*.*”* This particular tweet was categorized as positive content, because the human annotator judged the major sentiment as positive.

As summarized in Table 2, while the level of negative attitudes dropped from 80% to 52% after the official corporate apology, the level of positive attitudes increased prominently from 0.3% to 6.3%. A common thinking in crisis management practice is that one should not have high expectations for a praise or a sudden positive turn when a company publicly apologizes upon a crisis event. Rather a company can expect the public’s negative attitudes to assuage and become more rational. Our analysis supports this idea, as we demonstrated that the number of neutral tweets increased significantly from 20.1% to 41.7% after the apology. This result indicates the public apology in Domino’s Pizza event has lessened the amount of negative attitudes and increased the amount of neutral tweets.

### (Research Question 2) Do users’ sentiments toward a crisis event differ depending on the types of interaction they engage in social media?

Next we investigated if any of the factors related to the social network structure and the interaction types among users had an effect on a user’s sentiment. We consider the network structure, because the follow feature (i.e., social ties that connect users) is one of the most essential features of Twitter. To analyze the trend, we focused on the sentiment of users regarding the prank video (i.e., the bad news) and considered the tweets posted within 48 hours following the event (i.e., before the CEO’s apology) during April 13–15^th^. The tweets were categorized by the hourly time window they belonged based on posting times. We discarded time windows that contained fewer than 100 tweets from analysis and studied sentiments of tweets from 23 valid time windows.

To determine the effect of the social network structure or the interaction type on attitude changes, we performed sentiment analysis with the LIWC tool, taking into consideration the various types of user relationships. We analyzed the differences between the tweet sentiments of users who talked independently about the event and those who had one or more Twitter friends who mentioned the same event. The former group was labeled “isolated users,” as these users did not follow any other user who tweeted about the same event. They had independently talked about the topic. The latter group was labeled “connected users,” as these users were connected with at least one other Twitter user who tweeted about the same event.

The sentiments of the isolated and connected users were then compared using the two-sample t-test. No significant difference was found between the two groups in terms of both positive sentiment and negative sentiment (p>.05) (see Table 3). This result suggests that a social link did not play a role in propagation of sentiments for the Domino’s Pizza event. Rather, those who tweeted about the Domino’s event had similar sentiments, whether they were linked to one another or independently talked about the event.

**Table 3:** Statistics for the isolated and connected Twitter users.

	# of tweets	# of RTs	# of mentions
Isolated users	2,718	243	684
Connected users	2,009	559	535
Total	4,727	802	1,219

Our results do not deny the high-level of assortativity in social networks, as previous studies have found that happy or sad individuals form clusters in the offline contact network [33] and in the online contact network [17]. In contrast, our results suggest that while there may be high homophily in the general moods of those users connected in a social network, the mood does not need to depend on social distance if it is about a public event. For example, users may feel collectively sad about natural disasters like a tsunami or an earthquake regardless of their social distance. Similarly, in the Domino’s Pizza incident, typical individuals collectively felt disgusted about the prank video, regardless of their social hops.

**Fig 2:**
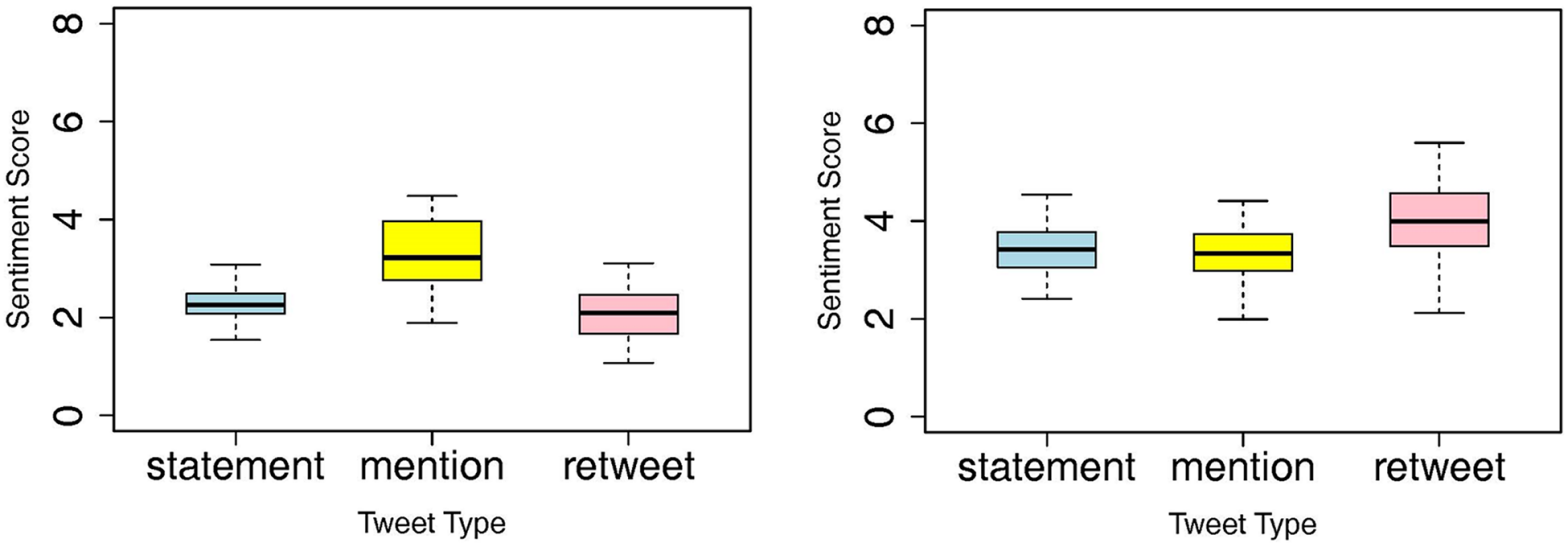
Average (A) positive and (B) negative sentiment scores of different tweet types (statement, mention, and retweet) with their standard deviations within the first 48 hours of the Domino Pizza's crisis, prior to the CEO's public apology.

We also investigated whether different forms of user interactions on Twitter, like retweets and mentions, had any effect on one’s sentiment toward the event. Fig 2 shows the average positive and negative sentiment scores among different interaction groups. “Statement type” refers to the Twitter users who independently talked about the Domino's incident, without interacting with other users via mention (@***) or retweet (RT) functions of Twitter. Analysis of variance revealed that retweets had higher negative sentiment scores than tweets without any interaction tweet (i.e., statement) (p<.05). This suggests that as the prank video was retweeted on Twitter, more users added negative comments. Whereas retweets had a stronger negative valence compared to the statement tweets, mentions had a stronger positive valence than the statement tweets (p<.05). This difference suggests that when users discuss bad news with others using the mention function on Twitter, they used less negative words than if they simply forwarded a tweet to others through the retweet function.

Next, we chose users who posted multiple tweets (i.e., three or four tweets) on the crisis event and grouped them into four types: (1) the statement group, who had posted only statement tweets; (2) the retweet group, who had posted at least one retweet; (3) the mention group, who had posted at least one mention tweet; and (4) the mixed group, who had posted at least one retweet and one mention tweet, respectively. For example, if a user had posted his or her personal thought (i.e., a statement tweet) on the Domino’s pizza event two times and retweeted another user’s tweet once, he or she would be classified as the retweet group.

**Fig 3:**
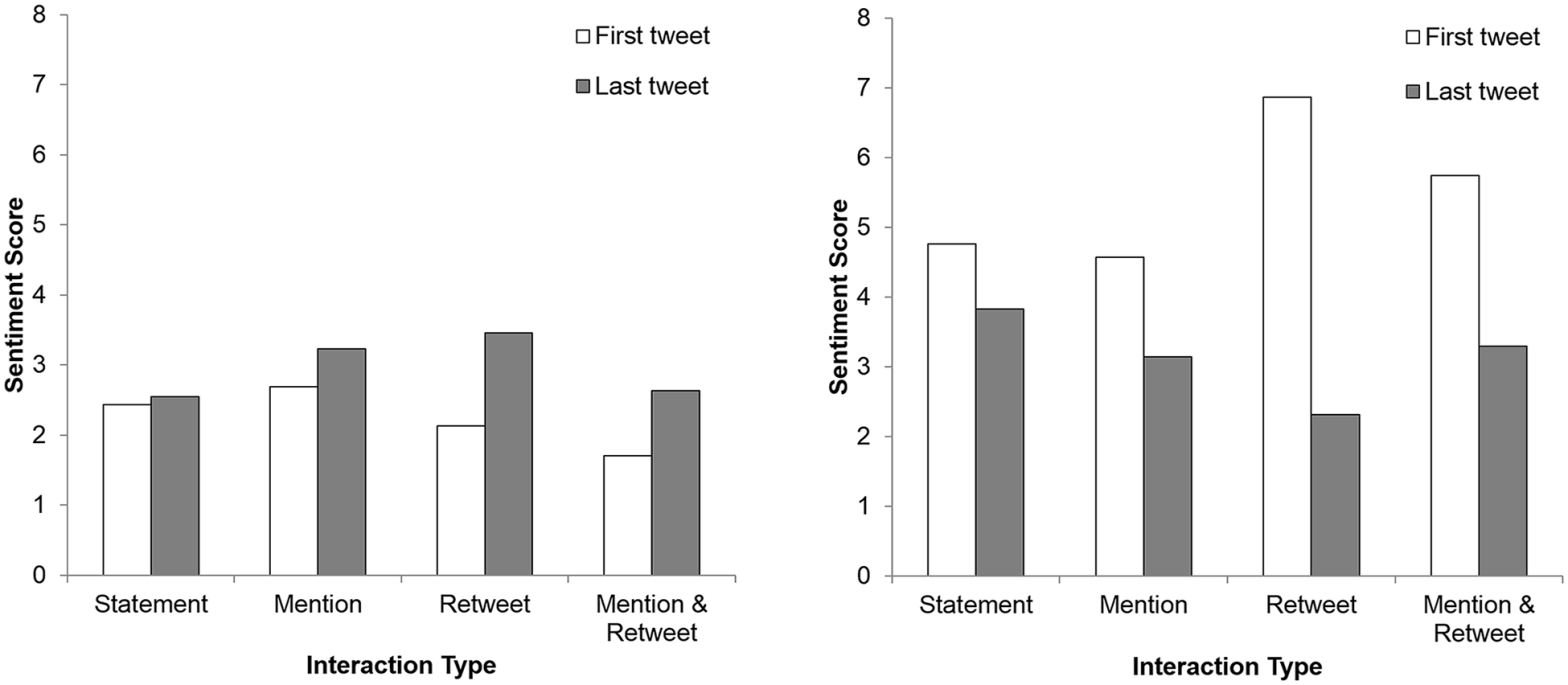
Average sentiment scores for four repeated interaction types (statement, mention, retweet, and mention & retweet) within the first 48 hours of the Domino Pizza's crisis, prior to the CEO's public apology. White bars and black bars indicate average sentiment scores of the first tweet and the last tweet, measured by Linguistic Inquiry and Word Count, respectively.

Then, to assess the effect of the four interaction types on users’ moods, we estimated the changes in sentiment scores from the first tweet and to the last tweet for each user. Fig 3 shows the trend of sentiment changes for the four interaction types. The level of negative sentiment decreased and positive sentiment increased as users conversed more about the topic. Those who posted only statement tweets (i.e., those who did not interact with other users in discussing the Domino’s case via mentions or retweets) exhibited the least amount of change in sentiments, in comparison to users who had interacted with others (i.e., the retweet group, the mention group, and the mixed group).

### (Research Question 3) What are the types of social media response upon a corporate crisis and how do they spread depending on the type?

In addition, we examined the temporal and topological features in the spread of Domino’s news. To identify and categorize the types of tweets, we manually observed the URLs embedded in the tweets. A majority of tweets (63%) contained a URL, and we considered those tweets containing a URL that appeared at least 10 times during the observation period. 90 distinct URLs were found in this fashion, where the most popular URL appeared 561 times. After manual inspection, we categorized these URLs into three representative groups as follows:

1. Bad News: 24 URLs on the prank video belonged to this group, which either contained a web link to the YouTube video or other web articles on the prank video, for instance, “U need 2 look @ this especially if u eat at domino’s http…” and “Be careful before ordering Domino’s: http…”
2. Apology News: 15 URLs on the apology video belonged to this type, which either contained a web link on the public apology, for instance, “Domino’s President responds http…” and “Excellent 2-min video on YouTube from Patrick Doyle. Congrats on this move http…”
3. Commentary: In the occurrence of a crisis, journalists, crisis management consultants, and consumers publish numerous articles with their perspectives on the incident. In such cases, Twitter is often used as a medium for these individuals to advertise their articles via sharing web links. 44 URLs hence belonged to this content type, for instance, “Apropos to the #dominos fallout, How to weather a #twitter storm http…” and “RT @briansolis: The Domino’s Effect http…”

Seven out of the 90 URLs could not be categorized to any of these three content types, and hence were eliminated from analysis. For each content type, we examined the social network structure of the involved users by testing if these individuals were connected through the follow link relationships. A large proportion of users from each of these networks were found to be connected to one other by Twitter’s follow links, and these users formed one large weakly connected network. Some users, however, were not connected to any other users who talked about the event (i.e., singletons), whereas others formed their own small networks and had no connections with the majority of users in the largest connected component.

Table 4 displays the structural characteristics of the bad news, apology, and commentary networks including the number of URLs, the number of tweets, the number of users who posted the URLs (whom we labeled as “spreaders”), the median number of Twitter users who follow these spreaders, and the distinct number of total followers of these spreaders (whom we labeled as “audience”). Audience represents the maximum number of possible users that received URLs on the Domino’s event via Twitter.

**Table 4:** Spatial characteristics of the twitter users who spread web links to the bad news, apology video, and commentary, respectively.

Type	# of URLs	# of Tweets	# of Spreaders	# of Median followers	# of Audience
Bad News	24	2,230	2,078	169	2,204,175
Apology	15	771	707	351	542,161
Commentary	44	1,641	1,608	367	4,706,032

A large number of users posted URLs on the Domino’s Pizza event. These users participated in distributing the prank video (n = 2,230) more than in distributing the apology video (n = 771) or commentaries (n = 1,641). Nevertheless, the median number of followers was the smallest for the prank network (n = 169), suggesting that the bad news (i.e., the prank video) was shared by less connected users in comparison to those who spread the apology news or commentaries. A few spreaders in each network owned disproportionally large number of followers and, as a result, the audience size was three orders of magnitude larger than the spreader size, reaching nearly 0.5 to 5 million Twitter users (Table 4).

The structural dissemination pattern has three implications: First, social media users give attention to public events at the onset of a crisis rather than at the end, which is when companies typically respond. That is, the bad news network has the greatest number of spreaders and tweets in comparison to the other networks, apology news and commentary (see Table 4).

Second, although the prank video had greater popularity than the apology or commentary videos, the median numbers of followers for both the apology and commentary networks were far greater than that for the bad news network. Therefore, we assume that although potential consumers or the average Twitter user may give more attention to the prank video (i.e., the crisis), influential individuals on social media such as journalists, marketing consultants, and power bloggers care more about how companies or the public respond to the crisis. These experts, who have greater degree of connection than those who just spread bad news, are the ones disseminating corporate responses and commentaries. As the apology and commentary networks had denser connections than the prank network did, this study showed the power of influence of the former groups. These experts are more likely to share their opinions and perspectives than information sharers, who only deliver the original news.

Third, crisis management consultants often say that bad news is shared more frequently than good news. The current study supports this general claim. Considering the mere number of total audience and the number of users who talked about the event (i.e., spreaders), one spreader disseminated the prank video to on average 1,061 (2,204,175/2078) people, whereas one spreader disseminated the apology video to 766 (542,161/707) people (Table 4). This vast difference in the number of spreaders for the prank video and apology video implies that an average social media users indeed deliver bad news more frequently to friends than they do so for good news.

### (Research Question 4) How do the tweets of different types (i.e. bad news, apology, commentary) diffuse in Twitter?

Since Web links are discovered at various rates based on their topics, we studied how the tweets containing an identical URL disseminated to users in Twitter. For this, we considered that a piece of information disseminated from user A to user B, if and only if (1) B follows A on Twitter, and (2) B posted the same URL only after A did so. Then the dissemination time for a piece of information to cross a social link is calculated as the difference in times between the tweet posts of A and B. In case a user had multiple sources, we selected the user who posted the same URL the latest as the source.

**Fig 4:**
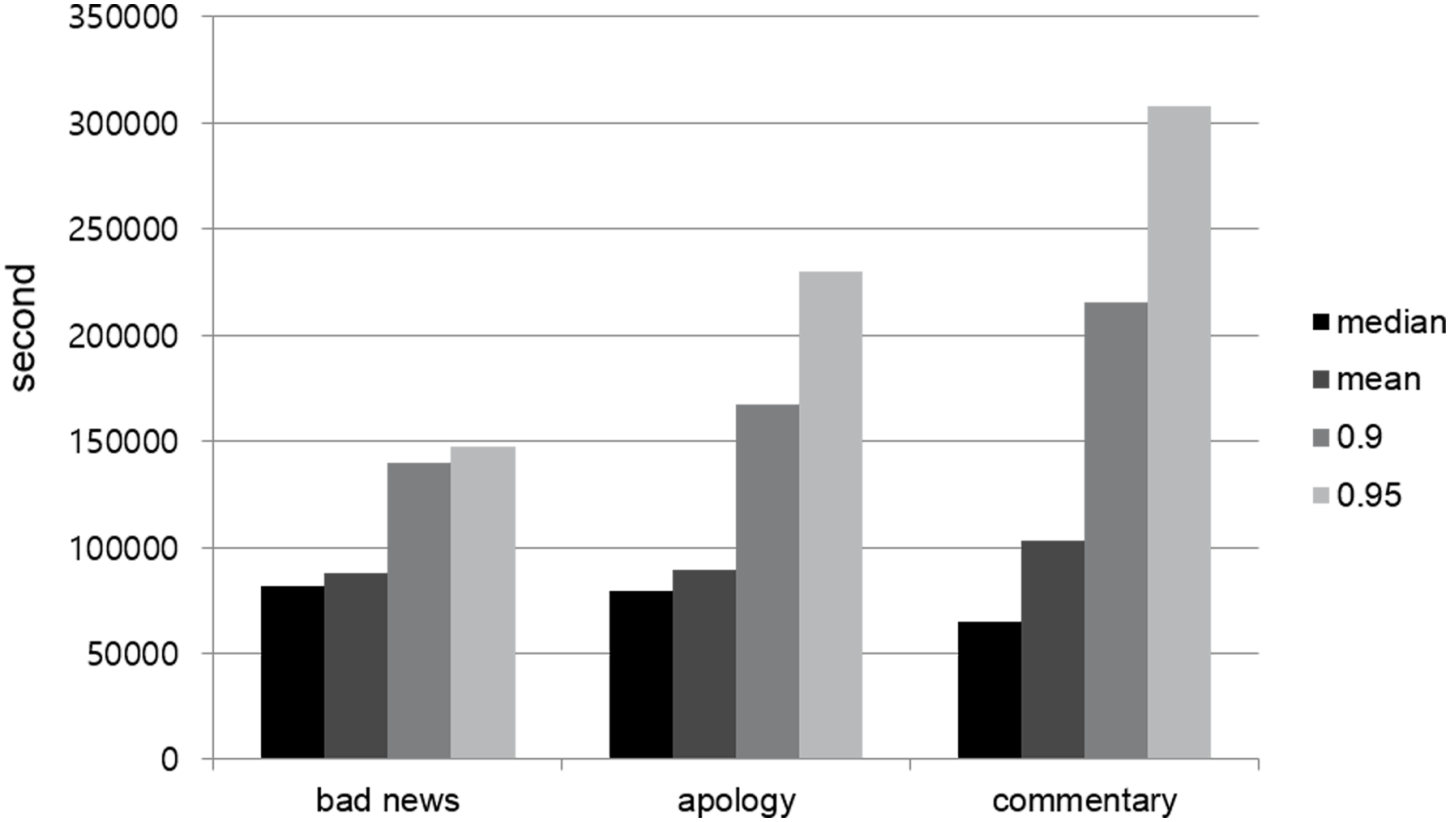
Cumulative distribution function of social diffusion times within Twitter.

As shown in Fig 4, we found that social diffusion times varied widely according to the type of news. The bad news took the longest time to diffuse over a social link with the median time of 81,835 seconds, compared with the apology and commentary news that took 79,163 and 65,212 seconds, respectively. The apology and commentary news took a shorter time to diffuse than the bad news, possibly because the bad news spread first to wider audiences and the apology and commentary news are about the bad news.

Based on the 95th percentile values, the spreading rate of the bad news decelerated after 147,816 seconds (or 1.7 days). On the other hand, the apology and commentary news spread at a fast rate for 230,335 seconds (2.7 days) and 307,922 seconds (3.6 days), respectively.

This temporal analysis has important implications. Compared to bad news, commentary tweets disseminated within Twitter for twice as long time and to a bigger audience. This is because, although slow, higher-degree users posted commentary tweets and over a longer period of time, ultimately reaching a wider audience. Given that the particular commentary was about how the corporate CEO handled the bad news by directly apologizing to the customers, it is interesting to observe that commentary had a longer-lasting effect than bad news.

### (Research Question 5) Upon a corporate crisis, to what extent do customers in social media show negative response that could impact product sales in future?

During a crisis event such as the Domino’s Pizza case, companies are concerned about damage in reputation, and more importantly, its damage in sales. As a matter of fact, during the first peak, the topical category related to negative purchase intention emerged with the three representative types of opinions. The first type was pretension, where some users showed that they would not eat at Domino’s from now on, for instance, ‘No more Domino’s at my house.’

The second type was persuasion, where some recommended others not to eat at Domino’s, for example, ‘*This Is Why You Never Eat Dominos Pizza* *http*:*//tinyurl*.*com/d22ubr*.*’*

The third type was perception, where some tweets confirmed people’s past negative purchase intent towards Domino’s, such as, *‘@TheDLC Due to their disgusting pizza*, *I also haven’t eaten at Domino’s pizza in about 20 years*. *Thanks for confirming my decision*!*’*

We investigated the level of negative purchase intention during the two peaks and found there was a significant drop from the first peak, which contained 131 such tweets (39.2%), to the second peak, which contained only 29 such tweets (7.3%). Finally, the qualitative analysis supported the idea that not all tweets about the CEO’s apology express positive sentiment. Among the 396 relevant tweets in the second peak, a total of 73 tweets (18.4%) talked about the apology, whereas 33 of them (45.2%) contained negative sentiment, for instance ‘*Very insincere response from Domino’s-* *http*:*//ow*.*ly/31mF*. *Compare to Jet Blue’s very sincere response 2 yrs ago-* *http*:*//ow*.*ly/31mV* *’* and 12 tweets (16.4%) were positive, for example, ‘*via @hollisthomases* *http*:*//bit*.*ly/2lZr8m* *kudos to Dominos for taking swift action via social media in response to the nasty employee videos’* while 28 tweets (38.4%) were neutral, such as *‘Razor Report Blog*: *Update—Domino’s Responds*: *Patrick Doyle*, *President*, *Domino’s U*.*S*.*A*., *resp*.. *http*:*//tinyurl*.*com/csls5n*.*’* Interestingly, nearly half of the tweets contained negative sentiment, even when they were about apology. Nonetheless, a little more than half of the tweets about the apology news were not negative, with 38.4%of them being neutral and 16.4%, positive.

### (Research Question 6) What is the role of a corporate representative account in social media upon a crisis and who communicate with the corporate account?

To answer this question, we collected a total of 456 tweets that mention Domino’s Pizza official Twitter account, @dpzinfo. Among them, 10 tweets were excluded, because they were either irrelevant to the Domino’s Pizza crisis or in foreign languages. We examined 446 relevant tweets that mentioned @dpzinfo and conducted qualitative analysis on them: 269 out of the 446 tweets (60.3%) were categorized as neutral, 131 (29.4%) as positive attitude, and 46 (10.3%) as negative attitude. The level of negative sentiment was significantly low, when compared with the first peak right after the crisis event (79.6%) as well as the second peak right after the official apology from Domino’s Pizza President (52%). This observation is consistent with the previously made finding from quantitative analysis in that mention tweets are less negative.

In each category, open coding was implemented to find major patterns:

1. Negative attitude includes sarcasm (e.g. *“Dominos opens a Twitter account to explain that they have not really added 'boogers and snot' to their toppings*. *@dpzinfo”)*, criticism (e.g. *‘sorry patrick doyle*, *president of dominos usa*, *but this just really doesn't count as an apology*: *http*:*//bit*.*ly/4ADzh1* *@dpzinfo’*), and future intention not to visit or purchase Domino’s Pizza (e.g. *‘@dpzinfo That video was gross*!! *I used to like Dominos but I won't order there again[m]*. *Sorry guys*. *Press felony charges on them*!!*’*).
2. Positive attitude includes positive evaluation about Domino’s crisis responses. Examples are *‘@dpzinfo Great job*, *Dominos*! *Thanks for the openness’*; *‘@dpzinfo Patrick Doyle's YouTube response to Dominos brand crisis VERY good*. *This is like Web 2*.*0 Tylenol*. *Case studies will be written*.*’*
3. Neutral attitude includes informational tweets. 163 out of 269 neutral tweets (61.0%) simply described Domino’s Pizza’s responses in social media. They were either introducing Domino’s Pizza’s official Twitter account (e.g. *‘Domino's created a Twitter account today as part of their Crisis Communications strategy over those videos*. *Very Interesting*. *@dpzinfo’*) or the President’s YouTube apology (e.g. *‘@perezhilton RT @dpzinfo Patrick Doyle*, *President of Domino's USA responds to the #dominos videos*. *http*:*//bit*.*ly/T0QXc*’). Among 163 tweets that neutrally described Domino’s Pizza’s responses, 50 (30.7%) tweets were featuring two of the popular original tweets: one by @briansolis (e.g. *‘RT @briansolis Domino's now on Twitter (@dpzinfo) responding to questions re*: *The Domino's Effect* *http*:*//poprl*.*com/0PLT* *’*) and another one by @chrispirillo (e.g. *‘RT @chrispirillo*: *Dominos PIzza responds to video WITH video*: *http*:*//bit*.*ly/4ADzh1* *- @dpzinfo is on Twitter*, *too*.*’*)

We observed that when Twitter users directly mentioned Domino’s Pizza’s official Twitter account, they exhibited a significantly low negative sentiment (11%), and the neutral (59.9%) or positive sentiments (29.1%) were much higher than in other cases. Among the tweets with neutral and positive sentiments, the majority of them were related to the Domino’s crisis response via social media rather than the bad news itself.

Then, we examined who those users were. A total of 378 distinct Twitter users participated in direct conversation with a total of 1040 tweets during the period and 420 tweets (40.4%) mentioned @dpzinfo. Among 378 accounts, 48 accounts posted five or more tweets during that period and showed active participation. In December 2012, we examined all of these 48 accounts to identify their public profiles and found that seven accounts no longer existed, one account was in a foreign language, and one was a protected Twitter account. For the remaining 39 Twitter accounts, based on their Twitter public profiles, 26 (66.7%) were practitioners in public relations, marketing communication, or media related industries, where these users described themselves with the following terms in their profiles (along with the number of times each keyword appeared in Twitter profiles): social/media/social media/new media (9), public relations(PR) (13), marketing/marketer (9), communication/communicator/ comm (6), Twitter/Google (3), blog/blogger/blogging (4), digital (6), journalist (5), and advertising (1). On 10 and 11 January 2013, we investigated the number of followers of these practitioner Twitter accounts to determine their overall influences. They were varied from 606 (@StevenBrisson) to 58,174 (@MarkRaganCEO), and the average number of followers was 7011, which was much larger than the typical user in Twitter.

The practitioners in marketing, PR, and media industry often use Twitter as a personal brand marketing or as a self-promotion channel to show their expertise to their followers to get more attention from audiences and potential clients and to build their presence online. Marwick and boyd [34] called it as ‘micro-celebrity’ technique, borrowing the concept from Senft [35]. According to Marwick and boyd [34], these kinds of practitioners have strategic audiences in mind when they tweet and adopt ‘self-censorship’ techniques. For example, they keep current and potential clients as strategic audiences and show their professionalism rather than personal feelings. In Domino’s Pizza crisis, the practitioners acted accordingly by minimizing personal negative sentiments towards the company, likely because Domino’s Pizza could be a potential client as well. At the same time, it was likely that they acted the role as ‘micro-celebrity’ by showing evaluation of crisis responses or by introducing the updated situations.

## Discussion

Here we report a case study on the real-time interaction behaviors of Twitter users during the 2009 Domino’s Pizza Crisis using both computerized sentiment analysis and in-depth qualitative analysis methods. We have analyzed a total of 20,773 tweets by 15,513 users that are relevant to the Domino’s Pizza incident from Twitter to assess the influence of bad news (i.e. the prank video) and public apology by the CEO on Twitter users’ sentiment and the direct communication patterns between individuals and corporate accounts during the crisis.

In this study, we made several key observations regarding the Domino’s crisis case in 2009. First, corporate executive’s public apology via social media significantly and immediately lowers the amount of negative sentiments in Twitter and also increases the level of factual tweets compared to opinion tweets, even though an increase in positive sentiments is marginal. Second, mention tweets are mostly positive and retweets are mostly negative, indicating that users engage in conversations (i.e. mentions) with more positive emotions than when they forward information to others (i.e. retweet). Third, public apology from the company significantly lowers the expression of negative purchase intention in tweets, which is possibly an important indicator of sales. Lastly, a large fraction of Twitter users who directly talk to the corporate account in a crisis situation are practitioners in PR, marketing, and media, who are the commentators from the earlier analysis.

### The audiences’ psychological model during and after an organizational crisis and response

One of the most dominant theories used in crisis communication research literature is Situational Crisis Communication Theory (SCCT) by Coombs and Holladay [36]. The theory demonstrates, from the organizational perspective, that the process through which organizations’ crisis responses is influenced by different crisis situations. Fediuk, Coombs, and Botero [10] expanded the SCCT and revised to suggest a stakeholder-focused theoretical framework, called the Stakeholder’s Cognitive Model (SCM), which describes the cognitive process of information handling during and after an organizational crisis.

The stakeholder’s cognitive model is based on the transgression-based crises such as organizational misdeeds or human-error accidents, where organizations have high responsibility for a crisis. The model has four phases [10]. The first phase is the trigger event process, where a crisis is triggered either as an organization violates stakeholders’ expectation or as stakeholders have perceptions of the violation. In Domino Pizza’s crisis, the prank YouTube video clearly violated stakeholders’ expectation as stakeholders expect that employees at a food company would take hygiene seriously and the company would have proper training and inspection system to keep the food safe.

The second phase is the evaluation process, where crisis stakeholders assess severity of a crisis event and crisis responsibility [10]. We reasonably assume that one of the key reasons for the shock and highly viral spread of the video is that stakeholders perceive it severely. Also, Domino’s Pizza had the responsibility from assuring that such event should not occur. Domino’s Pizza spokesperson Tim McIntyre later said ‘we got blindsided by two idiots with a video camera and an awful idea’ [37]. With these severity and responsibility in mind, finally, the President of Domino’s USA made a public apology via YouTube.

The third phase is the affective responses, which emphasizes the importance of understanding the moral outrage and any negative emotions in crisis such as anger or hostility [10]. The term affect is an interchangeable term with emotion. In Domino Pizza’s case, Twitter users expressed negative sentiments in their tweets especially during the time when the video circulated widely in the network.

The final phase is the outcomes, which according to this model the negative emotions of stakeholders in crisis situations would lead to two main outcomes: reputation damage and behavioral intentions [10]. In crises, as companies violate stakeholders’ expectations, people would have negative impression and experience with the responsible companies. The type of behavioral intention refers to revengeful actions by stakeholders, which are triggered by negative emotions for instance public complaints, and negative word-of-mouth. Domino’s Pizza clearly experienced both reputation damage and behavioral intentions. In one interview, Domino’s spokesperson Tim McIntyre said ‘Even people who’ve been with us as loyal customers for 10, 15, 20 years, people are second-guessing their relationship with Domino’s, and that’s not fair’ [37].

Among the existing crisis communication literature primarily focusing on SCCT, the stakeholder’s cognitive model is quite unique in that it is audience-centered. This model is potentially more useful as in the sphere of social media—where users have more power than in the traditional mass media, yet it has not been tested for crisis communication in social media. We should admit that this model provides us with hypotheses and suggestions in this study. Furthermore, the research questions covered in this study may be useful in testing particularly the affective responses and outcome phases of the stakeholder’s cognitive model.

Since conducting case studies as a scientific research method can help better understand, test or refine existing theories, and develop new theories [38], the current study provides us with an opportunity to revisit the stakeholder’s cognitive model [10] in the context of crisis communication on Twitter. This study demonstrates how negative attitudes (i.e. affective responses) create negative purchase intentions (i.e. outcomes). However, the current study suggests that the original stakeholder’s cognitive model has some limitations to directly apply to crisis communication in social media. First, while the original model focused on the affective (emotional) responses, audiences responded with mainly three aspects in a crisis situation: social, cognitive, and affective. In Domino’s case, Twitter users or audiences participated in the discussion (i.e., social), simply delivered facts (i.e. cognitive), and showed both positive and negative sentiments toward the event (i.e. affective). Second, the original model does not consider how the stakeholder’s cognition could be influenced by the Corporate Crisis Responses (CCR), yet the Domino’s case exemplified how corporate crisis responses possibly influence the audiences. Third, the audiences are influenced by how the traditional mass media and social media evaluate the crisis responsibility and the severity of damage of a company before making their own judgments. Fourth, the audiences consider the following in evaluating the corporate crisis responses: spokesperson (source), media (channel), contents (message), and timing (response). Fifth, reputation damages and future intentions could be either accelerated or decelerated depending on how appropriate corporate crisis responses are.

**Fig 5:**
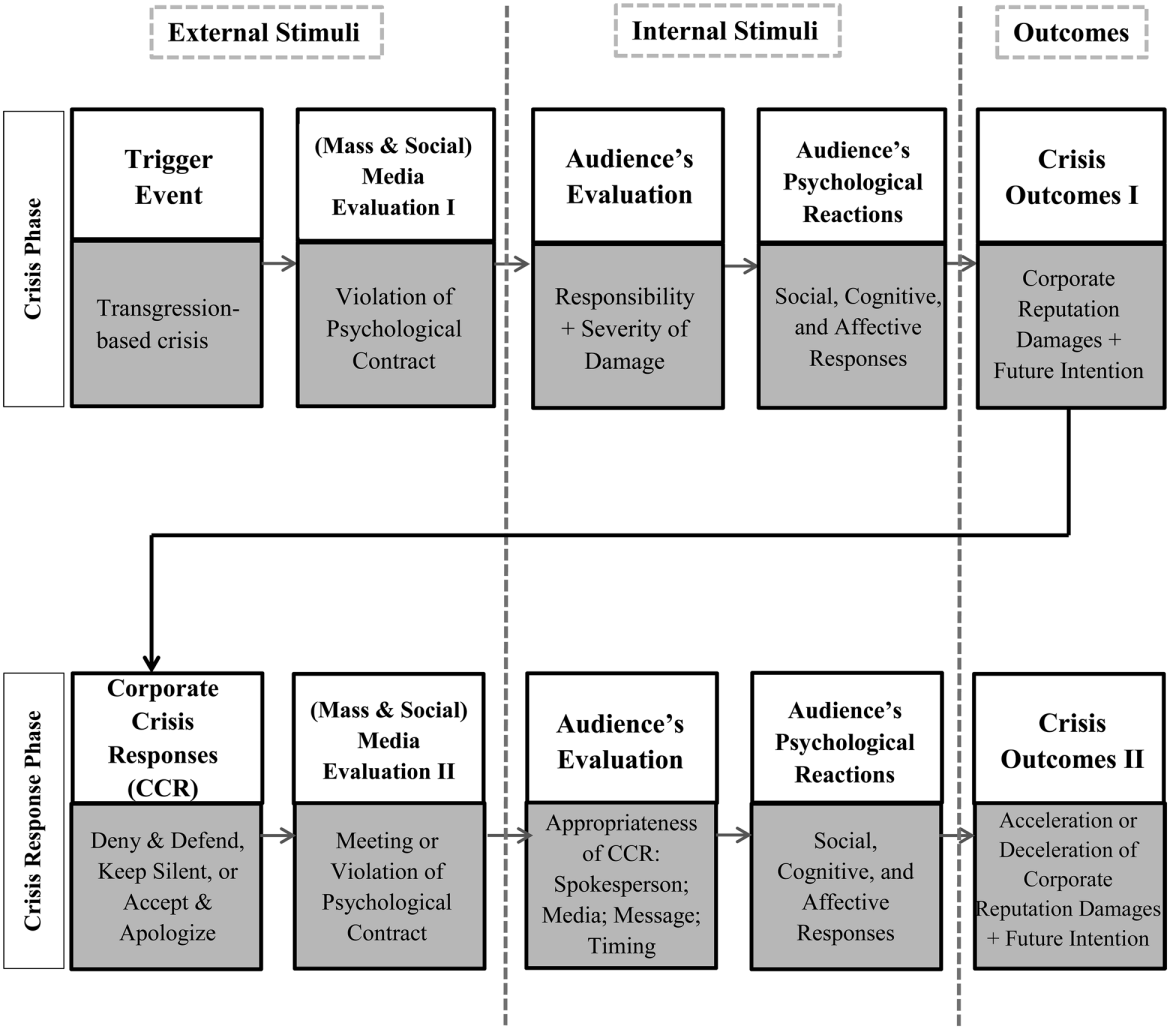
The audiences’ psychological reaction model during and after an organizational crisis and response. Revised from the stakeholder’s cognitive model for information processing during and after organizational crises by Fediuk, Coombs, and Botero in 2010. The model shows how an individual is psychologically influenced by external stimuli (crisis trigger event, corporate crisis response, media evaluations), processing the external stimuli internally (evaluations and psychological reactions), and shaping a crisis perception and future behaviors (crisis outcomes). The arrows are directions of the psychological processes and the two dotted vertical lines were drawn to show the boundaries of each of the three areas (external and internal stimuli and outcomes). The two phases refer to how crisis-triggering event and accordingly crisis responses are being processed both inside (internal stimuli) and outside (external stimuli) of an audience's mind.

Considering the above listed limitations of the original model, we propose a revised version of the stakeholder’s cognitive model that could be used in crisis communication (Fig 5). Our model has two major phases, where the model starts with the *Crisis Phase*, a step that describes triggering of an event by a transgression-based crisis. A crisis trigger event would first be evaluated either or both by the mass media and social media, who identify that an organization violated psychological contract with its audience (i.e. media evaluation). Then, the audience evaluate the responsibility and severity of damages (i.e. audience evaluation), and psychologically react to the crisis in three ways: social, cognitive, and affective (i.e. audience’s psychological reactions). These audience reactions would have impact on the corporate reputation damages and future purchase intentions (i.e. crisis outcomes).

The next phase is called the *Crisis Response Phase*, where the responsible companies react by taking any of the three responses: deny and defend, keep silent, or accept mistakes or wrongdoings and apologize (i.e. corporate crisis responses). The corporate responses initiate another series of processes. The mass media and social media evaluate the corporate crisis responses and judge whether they meet or violate the psychological contract with the audience (i.e. media evaluation). Audiences also evaluate whether the corporate crisis responses are appropriate or not considering the source, media, message, and timing (i.e. audience’s evaluation). After the evaluation, audiences show social, cognitive, or affective reactions (i.e. audiences’ psychological reactions). The corporate reputation damages and future intentions would be accelerated if the audience becomes even more negative with inappropriate corporate crisis response, while appropriate responses would lead to with positive or neutral responses from the audience and the damages could be decelerated (i.e. crisis outcomes).

One thing should be noted in this model. This model shows how an individual processes and reacts to crisis-related information. It has external stimuli (i.e. trigger event, corporate crisis response, and media evaluation) and internal stimuli (i.e. audience’s evaluation and psychological reactions). The mass media evaluation had previously dominant influences on audience’s evaluation and psychological reactions, but in the age of social media the audience’s evaluation and psychological reactions are reflected in online social community. According to the annual trust barometer study by Edelman [39], ‘a person like yourself’ (65%) was ranked as one of the top three, most credible spokespersons, after an academic or expert (68%), and technical expert in the company (66%). Compared with the same survey conducted in 2011, ‘a person like yourself’ significantly increased by 22% (from 43% to 65%), while the impact of other factors dropped largely.

### Limitations, suggestions and implications

We should admit that this study has some limitations. Among them, one limitation is at unclear causal relationship between public apology and sentiment change. While our analysis have shown that corporate public apology via social media lowers the amount of negative sentiment, this study could not quantitatively determine the influence of public apology on negative sentiment of Twitter users. We could not exclude the possibility that time also affects the change in users’ sentiment, as they tend to forget what happened or become less emotional over time. Another limitation is at the case study approach adopted in this study, which naturally limits us from observing general trends.

Admitting these limitations, we discuss findings from analyzing additional cases on corporate crisis situations accompanying CEO’s apology. The first crisis (Case 1 in S1 Appendix) occurred in South Korea [40] (S1 Appendix Additional Case Analyses), which is related to a large retailer E-mart mislabeling imported beef as Korean-beef and upsetting customers. The case revealed the following dynamics (Figure A in S1 Appendix): the level of negative sentiment increased prominently during the crisis, and then decreased significantly after the Twitter apology by Mr. Chung, the vice chairman. This dynamics has stark similarity with the Domino’s Pizza case. The analysis also identified a unique finding on the difference in sentiment changes; those who followed Mr. Chung on Twitter were more likely to show a lesser degree of negative sentiment after the apology than those who did not follow Mr. Chung prior to the event. This finding indicates that building trust and relationship prior to a crisis has a major impact in the effect of apology. In both crisis events by Domino’s Pizza and E-mart, the level of negative sentiment immediately dropped after the public apology of a CEO on social media. A complete dataset for this analysis can be found in S1 Dataset.

However, not all public apologies by a CEO via social media will lower the degree of negative sentiments. The Case 2 in S1 Appendix on KFC’s crisis demonstrates that even when a chief executive apologizes via social media after their mistake, negative sentiments will not immediately decrease and rather increase after the apology (Figure C in S1 Appendix). A possible reason for ineffectiveness of public apology via social media could be that social media users do not perceive certain public apologies as appropriate or public apologies were not propagated well. In this particular case, criticisms were raised because the CEO was smiling during the apology speech and tried to justify the failure.

In a third case study, we discuss a crisis event that was not followed by any official public apology by CEO via social media (Case 3 in S1 Appendix). The particular case is about a failure in customer service of United Airlines, where the airline mishandled luggage and a $3,500 guitar was found broken. This crisis went public when the customer posted a song on YouTube describing the event after failing to get any compensation for 9 months. The song provoked negative sentiments on Twitter, which started to decrease after seven days without any public apology via social media (Figure E in S1 Appendix).

These additional cases help us gain richer dynamics of real world events related to the effect of public apology upon crisis situations. It is very challenging to systematically control for the lack of the presence of a public apology in a real world and experimental settings, which future studies can tackle. Nonetheless, the current study contributes to by demonstrating the methodology to quantitatively and qualitatively measure public sentiments toward a crisis situation via social media and further by showing how negative sentiments upon a major crisis situation can change through different types of user interactions (e.g., mentions, retweets) and through a public apology by a CEO on social media.

As social media play a critical role in crisis situation, big data analysis becomes essential in better understanding how people react to bad news, disseminate the information with one another, and communicate with organizations and leaders. The current study quantitatively and qualitatively demonstrated the benefit of big data analysis in crisis communication research. Our finding on how different categories of information such as bad news and public apology lead to distinctive dissemination patterns in terms of temporal and topological features is novel.

Future research should consider other factors in analyzing social media crisis, for instance 1) characteristics of the crisis (e.g. technology- or environment-related crisis); 2) social media channels (Twitter or Facebook); 3) previous perception towards the company responsible for the crisis; 4) the history of the company in whether it make efforts to build relationship with customers before the crisis event; 5) timing and appropriateness of corporate crisis responses (denial or apologies); 6) the use of an official corporate account or a personal account like those of CEOs’ in the apology; 7) whether CEO apologized or not. Future studies should focus on one or more of the above factors in studying crisis communication in social media.

The findings of this study have the following practical implications. First, this study potentially gives a guideline for how companies proactively respond upon a social media crisis by identifying the specific cases that decreased the level of negative sentiments. Companies need to respond to the crisis event rapidly, for instance, by delivering a proper public apology. In addition, installing an official social media account and conversing with stakeholders may be helpful for reducing negative sentiments.

Second, this study identifies the role of commentators upon a crisis event, as these users hold high influence over other Twitter users (i.e. having many followers) and are keen on the topic (i.e. being practitioners in PR, marketing, and media). In the current study, their tweets stay and share (retweeted) for a relatively long period among the followers. The commentator group has the following characteristics: 1) they actively mention the company by either frequently mentioning the company or by being retweeted frequently; 2) they focus more on how a company responds to crisis, and they update their followers with the corporate responses and non-negative sentiments, either positive or neutral sentiments. For example, influential media strategists like @briansolis (who has 166,605 followers as of January 2013) and @chrispirillo (who has 121,960 followers as of January 2013) play a key role in spreading Domino’s apology news and their moves about the newly setup official Twitter account. The findings from the current study suggest two active insights for companies: 1) engage with active commentators on Twitter early upon the start of a crisis event; 2) in a crisis situation, companies should update the commentators on their responsive actions, because commentators hold high influence within Twitter.

In conclusion, the current case study clearly demonstrates sentiment dynamics of Twitter users in responding to corporate bad news and public apology of CEO and the critical role of official corporate account for managing negative sentiments through bi-directional active communication with users. Here we support the value of the famous claim that ‘Markets are conversations’ [41] especially in crisis situations—that conversation is a better crisis communication tool than keeping silence or enforcing a one-way conventional communication.

## Supporting Information

S1 Appendix Additional case analysis. (DOCX)

S1 Dataset Data from studies. (ZIP)
