# Supplementary material for: The Effect of Bad News and CEO Apology of Corporate on User Responses in Social Media
Source: PLoS One. 2015 May 7;10(5):e0126358. doi: 10.1371/journal.pone.0126358 (PMC4423858; doi:10.1371/journal.pone.0126358)
Supplement: S1 Appendix — (DOCX) [file pone.0126358.s001.docx]

**S1 Appendix. Additional Case Analyses**

**The effect of bad news and CEO apology of corporate**

**on user responses in social media**

Case 1. E-Mart CEO’s apology in South Korea

The crisis began on July 27^th^ in 2010, when a local government’s veterinary service announced that a regional store of E-mart, the largest retailer in Korea, labeled U.S. beef as produced in Korean [1]. In Korea, customers prefer domestic beef over imported products, and hence its price is more expensive. Following the incident, online users showed angry reactions towards the E-mart retailer through various social media including Twitter. Similar to the Domino’s Pizza event, a public apology followed quickly, only a day after the crisis began on July 28^th^. The CEO of E-mart, Mr. Byung-ryul Choi, made a public apology via Twitter, and so did Mr. Yongjin Chung, the Vice Chairman of Shinsegae Group (the mother company of E-mart), who retweeted Choi’s apology. Unlike in the Domino’s case, Mr. Chung had been a popular public figure and a business executive who had been active in Twitter *prior* to the crisis, owning 39,000 Twitter followers. In contrast, the president of Domino’s Pizza was not present on social media prior to the crisis.

**
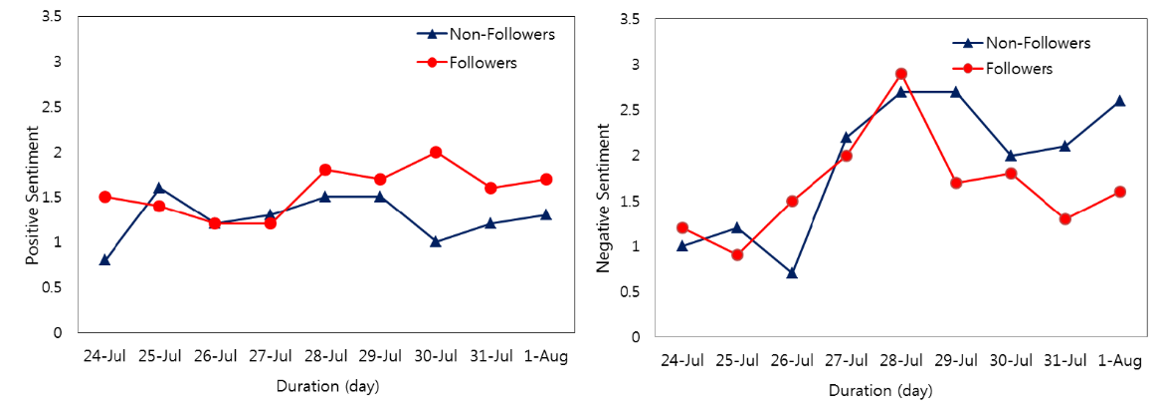
**

Figure A. Temporal evolution of the positive and negative sentiments (E-Mart, 2010)

For a qualitative analysis, we sampled relevant tweets from two peak times, on 27^th^ and 28^th^, which correspond to the time period right before (1^st^ peak) and after the public apology (2^nd^ peak). Given a small sample size, our focus was to investigate whether a consistent pattern emerges among various user types (i.e., whether users had followed Mr. Chung prior to the crisis event and whether they had mentioned Mr. Chung during the event). Table S1 shows that the level of negative sentiment decreased after the public apology. The level of positive sentiment, on the other hand, increased prominently in three out of four groups.

Table A. Qualitative analysis results of tweets (E-Mart)

|  | **The 1^st^ peak** | **The 2^nd^ peak** |
| --- | --- | --- |
| Neutral |  |  |
| non-followers | 2 | 6 |
| followers | 1 | 2 |
| non-mention | 2 | 2 |
| mention | 2 | 1 |
| Positive |  |  |
| non-followers | 0 | 0 |
| followers | 0 | 11 |
| non-mention | 0 | 5 |
| mention | 0 | 5 |
| Negative |  |  |
| non-followers | 23 | 13 |
| followers | 4 | 3 |
| non-mention | 10 | 5 |
| mention | 3 | 1 |

Case 2. KFC CEO’s apology in USA

On May 5th, 2009, KFC launched a grilled chicken giveaway campaign via The Oprah Winfrey Show. Viewers were told that they can visit Oprah.com for the next 24 hours where they can download a coupon for new grilled chicken menu items. However, many people were not able to download the coupon and some KFC restaurants did not honor the coupon [2,3]. This event led to an increase in the number of tweets from May 5^th^ (Fig. S2).

On May 7^th^, the CEO of KFC apologized via online video, offering a rain check program for angry customers. However, negative emotions were not decreased on Twitter and rather it increased slightly (Fig. S3). A possible reason for ineffectiveness of public apology via social media could be that social media users do not perceive certain public apologies as appropriate or public apologies were not propagated well. In fact the CEO’s apology was criticized: the CEO was smiling as he delivered the video apology and also tried to justify the failure by stating it is due to popularity of their menu rather than accepting the poor planning and coordination for the promotion [4].


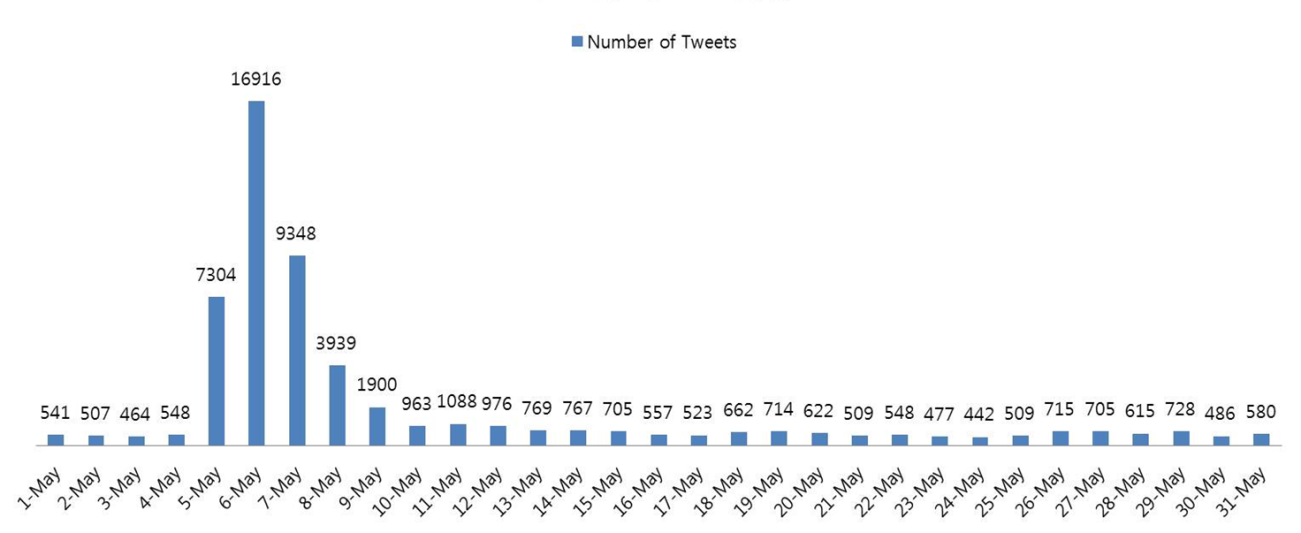


Figure B. The number of tweets over time (KFC, May 2009)


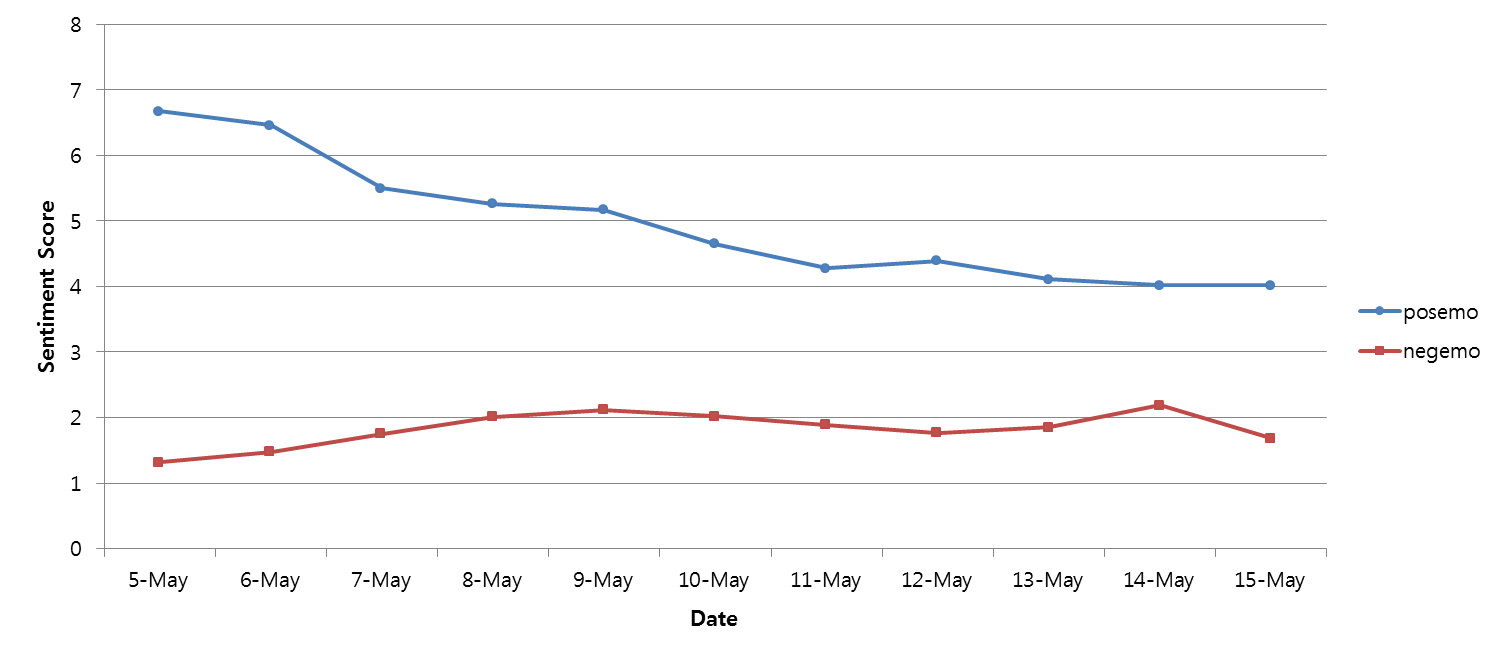


Figure C. Average sentiment scores (KFC, May 2009)

Case 3. United Airlines’ crisis in USA (without any CEO’s apology)

In March 2008, Dave Carroll, a professional musician, watched his $3,500 guitar was thrown away by a baggage handler of United Airlines. Mr. Carroll had to claim several times almost nine months but United Airlines rejected to compensate to repair the guitar. Mr. Carroll decided to write a song about his negative experience with the airline. He uploaded his song “United Breaks Guitars” with Youtube video on July 6^th^, 2009, and more than 500,000 viewed the video by July 9^th^. On July 8^th^, United Airlines personally called Mr. Carroll to apologize, but they did not use social media to publicly apologize, at least until July 13^th^ [5,6,7]

Figure S4 shows daily amount of tweets related to the incident in July 2009.

Between July 6^th^ and 13^th^, overall negative emotions are increasing while the overall positive emotions are decreasing in Twitter (Fig. S5).


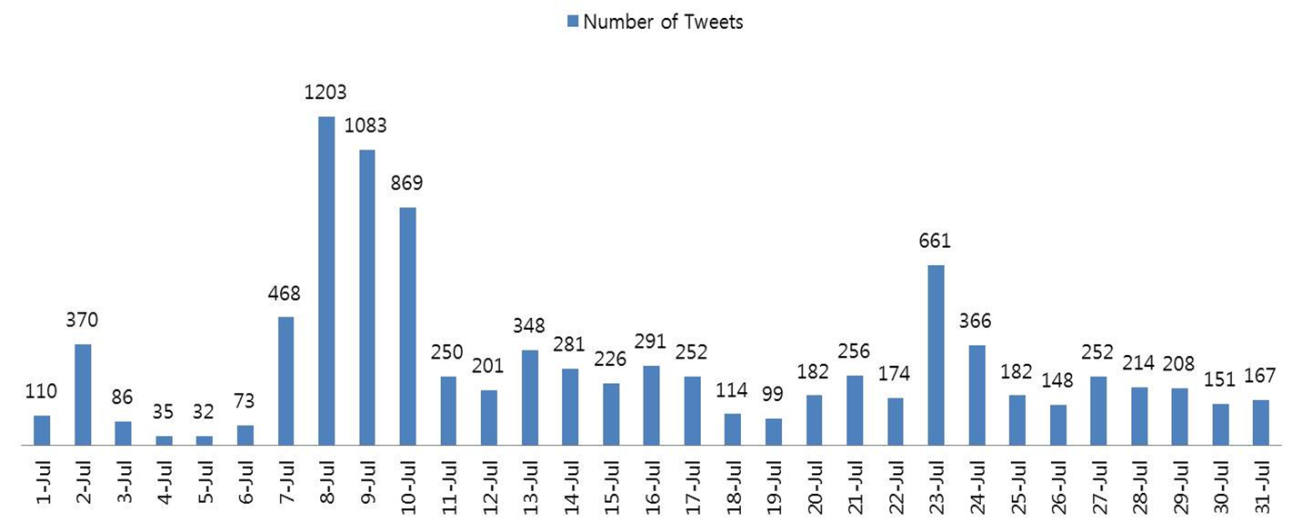


Figure D. Number of tweets (United Airlines, July 2009)


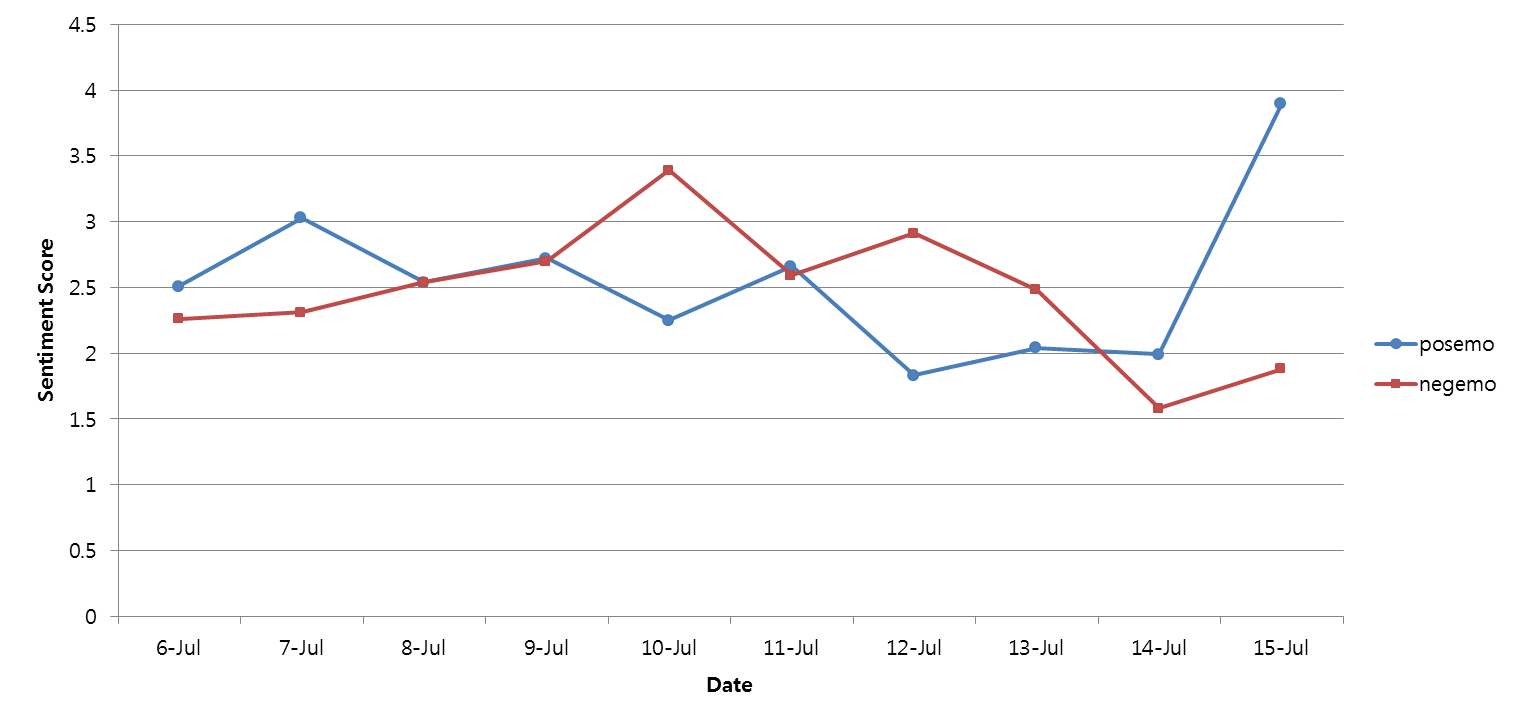


Figure E. Average sentiment scores (United Airlines, July 2009)

**References**

[1] Park J, Kim H, Cha M, Jeong J (2011) Ceo’s apology in twitter: A case study of the fake beef labeling incident by e-mart: Springer.

[2] KFC (2009) America has overwhelming response to Kentucky grilled chicken offer; KFC offers apology and rain checks for inconvenienced customers. Available: <http://www.kfc.com/about/news/2009/050709.asp>.

[3] France L (2009) Oprah coupon craze leaves KFC customers hungry for more. CNN.

[4] Walderich L (2010) The CEO apology: A new kind of brand booster? Available: <http://www.fastcasual.com/articles/the-ceo-apology-a-new-kind-of-brand-booster/>.

[5] UPI (2009) Singer's revenge on United: A hit song.

[6] Dave Carroll (2013) Personal Branding in the Age of Social Media: Dave Carroll at TEDxHoboken. Available: <https://www.youtube.com/watch?v=u-R-TeGMufA>.

[7] Greenfield D (2009) United Airlines Online Public Response to Dave Carroll YouTube Video: 9 Tweets. Social Media Today. Available: <http://www.socialmediatoday.com/content/united-airlines-online-public-response-dave-carroll-youtube-video-9-tweets>.
