# Supplementary material for: The Effect of Bad News and CEO Apology of Corporate on User Responses in Social Media
Source: PLoS One. 2015 May 7;10(5):e0126358. doi: 10.1371/journal.pone.0126358 (PMC4423858; doi:10.1371/journal.pone.0126358)
Supplement: S1 Dataset — (ZIP) [file pone.0126358.s002.zip › README.doc]

Twitter Dataset for PLoS ONE Paper by Kim et al. 
 

Contact Please contact Hoh Kim (hoh.kim@kaist.ac.kr) or Jaram Park (jaram.park@kaist.ac.kr) at the Graduate School of Culture Technology, KAIST.  
 
Data description We make all the data used in the study available, which we uploaded along with the revised manuscript. The numeric IDs included in the dataset have been anonymized following the PLoS ONE data policy. Adhering to the Twitter's data policy, the full content of individual tweets is not allowed to share. Given these constraints, we make the following information available to the public. The data below are sufficient to study dissemination structures of various information types discussed in the manuscript. 
(1) The list of anonymized tweet IDs, user IDs, and their posting times for the three conversation types (i.e., bad news, apology, and commentary information)
(2) The linkage or the follow graph information among the participating users

Description of the full Twitter dataset and the network topology can be found from the following paper:  Cha, M.; Haddadi, H.; Benevenuto, F.; and Gummadi, K. 2010. Measuring User Influence in Twitter: The Million Follower Fallacy. In Int. AAAI Conf. on Weblogs and Social Media (ICWSM). 
 
Data format 

Domino
List of user and tweet:  
Location: Domino/User_Tweet/*_uid_tid_time.txt 
Format: Tweet Id	User Id	Time
Example: 1529339590	14748004	2009-04-15 23:26:01
 
Network information (edge): 
Location: Domino/Network_Topology/*_edges.txt 
Format: userid_source userid_target (userid_source follows userid_target) 
Example: 10203002 1036761 
 
Network information (node): 
Location: Domino/Network_Topology/*_nodes.txt 
Format: userid  
Example: 10203002 

E-mart
List of user and tweet:  
Location: Emart/emart_tid_uid_time.txt
Format: Tweet Id	User Id	Time
Example: 19378626193	85084832	Sat Jul 24 00:01:33 +0000 2010

Follower list of Yongjin Chung
Location: Emart/followers_yjchung.txt 
Format: user id 
Example: 10203002
